# Baseline task performance predicts mini-mental state examination improvement after individuals with dementia practice an N-back task with bilateral transcranial direct current stimulation

**DOI:** 10.1371/journal.pone.0332372

**Published:** 2025-10-29

**Authors:** Carlos Roncero, Alex Popov, Bruna Seixas-Lima, Durjoy Lahiri, Fatemeh Abnavi, Howard Chertkow

**Affiliations:** 1 Baycrest Academy for Research and Education, Baycrest Centre for Geriatric Care, North York, Ontario, Canada; 2 Lady Davis Institute for Medical Research, Jewish General Hospital, Mcgill University, Montréal, Québec, Canada; 3 Division of Neurology, Department of Medicine, Queen’s University, Kingston, Ontario, Canada; 4 Audiology and Speech-Language Pathology Program, School of Rehabilitation Sciences, University of Ottawa, Ottawa, Ontario, Canada; Aalto University School of Science and Technology: Aalto-yliopisto Insinooritieteiden korkeakoulu, FINLAND

## Abstract

**Introduction:**

This research explores the relationship between baseline performance in dementia patients and its impact on the effectiveness of transcranial direct current stimulation (tDCS) for improving executive function. Past research suggests that those participants who are more impaired at baseline will be those who then demonstrate the greatest improvements with tDCS. In addition, we examined if bilateral or unilateral stimulation would be more effective for improving the general level of cognition of these participants.

**Materials and Methods:**

The study involved 24 participants, with 18 diagnosed with Alzheimer’s disease (AD) and 6 with Frontotemporal Dementia (FTD). These participants engaged in three rounds of N-back task training sessions, each separated by two months. The sessions included three types of stimulation: 4mA unilateral stimulation targeting the left Dorsolateral Prefrontal Cortex (DLPFC), 4mA bilateral stimulation targeting both the left and right DPFC, and sham stimulation. Additionally, a control group of elderly individuals without dementia was used to establish a benchmark for the normal average response time (RT) of the administered N-Back task. Evaluations, comprising of the N-Back task and the Mini-Mental State Examination (MMSE), were conducted before stimulation, at the final session, and two weeks post-stimulation in all rounds.

**Results:**

Participants were retrospectively allocated into those whose baseline N-Back response times (RTs) were found on average to be within the normal range versus those who performed more slowly than the normal range. For the group that performed within the normal range, no significant improvement was observed on the N-Back task after a series of sessions involving active tDCS. In contrast, the participants with slower than normal RTs at baseline demonstrated improvement both on the N-Back task (faster times) and the MMSE (higher scores) after receiving active tDCS, although accompanying improvement on the MMSE task was restricted to the condition where participants received bilateral tDCS.

**Conclusion:**

This study replicates a pervasive finding in the tDCS literature: participants who perform worse at baseline often demonstrate the greatest improvements with tDCS. Furthermore, these results have clinical implications, in that tDCS may be most beneficial in individuals when clear deficits or impairments are present. Attempts to treat less severe participants might fail to show improvement with tDCS. Ultimately, baseline condition should be routinely considered when evaluating tDCS results.

## Introduction

Transcranial direct current stimulation (tDCS) has been explored as a means of improving brain function across a range of neurological and psychiatric conditions [1–3]. Results have been mixed according to the conditions studied, but even studies within a particular neurological condition such as dementia have yielded quite variable results [4,5]. Explaining these variable results is challenging because studies have used different experimental methods in terms of electrode placement, study task, target population, and stimulation intensity. Nevertheless, a particular pattern has often been reported: those participants showing the most notable baseline slowing or impairment on a task are most likely to demonstrate larger improvements in accuracy or response time (RT) on the task when it is targeted by tDCS. This pattern is widespread in the tDCS literature, with over 20 studies reporting the phenomenon [6–27]. Notably, except for Heinen et al. (2016) [19]—who observed similar effects using cathodal stimulation—all studies employed or reviewed anodal tDCS aimed at neural excitation. Habich et al. [28] remarked that tDCS seemed to be *“following the seemingly inherent principle of greater stimulation gains in individuals with lower baseline skills* (p.8).*”* Until now, however, this inherent principle is without a particular coined notation. For this reason, we will hereafter refer to this pattern of results as the R.I.S.E. principle: reduced initial performance stimulates enhancement. To be clear, the R.I.S.E. principle refers to the commonly observed result where it is paradoxically the individuals worse at baseline (i.e., reduced initials), that go on to have the greatest improvements on a given task (i.e., enhancements) after receiving tDCS. In this manuscript, we will first briefly review the literature to date that has reported results consistent with the R.I.S.E. principle. Next, we report our own study where results consistent with the R.I.S.E. principle were found in a group of people living with dementia, including Alzheimer’s Disease (AD) and Frontotemporal Dementia (FTD). These participants engaged in a study designed to compare unilateral and bilateral tDCS, but also reinforced a conclusion reached by past researchers: room for improvement is a prerequisite for tDCS enhancements, regardless of the form of tDCS given.

### Introducing the R.I.S.E. principle of tDCS

Reviewing a series of tDCS studies, Looi et al. [13] noted that the phenomenon tended to emerge in a categorical manner: low vs. high anxiety [11]; low vs. high visual short-term memory [14], with results often superior or present for one of the groups (typically the one deemed lower). Furuya et al. [15], for example, chose to administer tDCS to a group of untrained pianists, but also a group already highly skilled. Consistent with predictions, improvements were found only for the untrained pianists. Rosen et al. [17] provided a neurological explanation when discussing tDCS enhancements for jazz improvisation that were isolated to novices post-tDCS. They argued that experience may allow for brain networks to be so optimized that they impose a ceiling effect that prevents tDCS enhancements. This hypothesis also has the benefit of explaining past results noted by researchers who found superior results in older vs. younger participants [29], impaired vs. healthy impaired participants [30], and the absence of benefits in healthy young adults [31].

Some researchers have also explained the R.I.S.E. principle in terms of improved resting-stage functional connectivity [32,33]. For example, spectroscopy studies examining the neurological effects of tDCS [34–36] have reported that tDCS administration to the motor cortex resulted in decreased GABA levels, that coincide with better performance on behavior tasks (e.g., motor sequence learning). As GABA is involved in the maintenance of cortical excitation and inhibition [37], a decrease could indicate increased excitability in the targeted task-related brain regions. This increased excitability may in turn lead to the improved performance observed, as well as initiating processes of neuroplasticity and long-term potentiation that translate to continued improvement post stimulation [38,39]. tDCS has also been found to enhance oscillatory activity in gamma- [40], beta- [41], alpha- [42], and theta-bands [43], which are known to be impaired in mild cognitive impairment [44,45]. Finally, a recent study found evidence for increased metabolic rates in a person with frontotemporal dementia (FTD) after completing a series of tDCS sessions [46]. Thus, while tDCS enhancements (in terms of improved accuracy and decreased RT) may be absent for some populations, there may still exist therapeutic potential for individuals having conditions where cognitive excitation and relative neural bands are impaired.

### Presence of the R.I.S.E principle when tDCS is used for cognitive impairment

tDCS studies targeting cognitive impairment predominately involve placing an anode electrode over the left dorsolateral prefrontal cortex (DLPFC) while participants complete a task designed to tax working memory, as it involves abilities found to deteriorate with age (e.g., processing speed, attention, executive functioning) while less-fluid abilities (vocabulary, acquired knowledge) remain intact [47]. Consistent with the R.I.S.E principle, these studies often report selective enhancements among individuals with low baseline working memory abilities [13,18–20,]. Katz et al. [21], for example, reported that each standard deviation increase in the baseline measurement (*n*-back performance) was associated with a corresponding decrease in the tDCS enhancement observed. For this reason, research has progressively incorporated baseline performance into analyses by either forming dichotomous groups at enrollment or retrospectively, based on participants’ initial performance on specific tasks or their overall cognitive ability at baseline. Alternatively, baseline scores on a specific task have been utilized. For instance, three studies [23–25] uniformly reported participants with the lowest baseline performance on the specified training tasks exhibited the most significant improvements in general cognition.

tDCS studies involving people with dementia have also reported the R.I.S.E. principle [16], but we know of only one study to date where the researchers sought to improve cognition by administering a training task with tDCS. Rodella et al. [27] found that younger patients with lower MMSE scores were those who went on to show a marked increase. The authors reiterated the argument for the R.I.S.E. principle in tDCS, especially noting its explanation for negative tDCS results caused by ceiling effects. More specifically, the R.I.S.E. principle can be seen as related to the ‘compensation account’ hypothesis [48], which predicts that people who have achieved a good level of cognitive functioning will in turn have a reduced range of potential improvement.

Due to the relative rarity of tDCS cognitive training studies involving people with dementia (AD, FTD); especially those examining baseline performance as a mediator of the results found, we designed our own working memory training program with tDCS that was expected to improve overall cognition. Considering the prevalence of the R.I.S.E. principle in past studies, we expected that individuals performing in the normal range on the training task would fail to benefit from tDCS. More specifically, we expected that only participants performing below normal on the task at baseline would demonstrate an improvement whereas higher performing participants would be less likely to show any improvement change due to a ceiling effect that prevents observable improvements.

### Unilateral vs. bilateral stimulation

As previously discussed, most tDCS studies have administered tDCS to the left hemisphere, but both hemispheres are predicted to have some damage once individuals receive a diagnosis of FTD or AD. Therefore, it stands to reason that stimulation to both hemispheres might be more effective than one hemisphere alone. Beyond the simplistic notion that two is better than one, executive function has been found to be instantiated bilaterally in the prefrontal cortex [49]; more specifically, the frontoparietal network (FPN): a large-scale brain network involved in sustained attention, complex problem-solving, and working memory. Anatomically, the FPN is primarily composed of the bilateral dorsolateral prefrontal cortex (the rostral lateral and dorsolateral prefrontal cortex, especially the middle frontal gyrus), but includes the posterior parietal cortex, which is involved in cognitively demanding tasks and damaged in cases of AD and FTD [50,51]. Also, studies have suggested a non-response to tDCS may be related to an overly small electric field size generated by the tDCS administered [52]. Thus, a montage that generates a wider electric field across both hemispheres may be superior to a unilateral one.

### Present study

This study was conducted with several examinations in mind. First, whether performance on a task given during stimulation (the N-Back task) would improve more when practiced with concurrent tDCS compared to a sham (placebo) condition. Second, we predicted that results would strongly be impacted by the performance level of the participants, such that only those participants showing impairment below the range of normal on the N-Back task would also demonstrate a tDCS effect. Third, we predicted that those participants who demonstrate a tDCS effect on the N-Back would also demonstrate an improvement in general cognition, with a proxy being the improvement in the Mini-Mental State Exam (MMSE) [53]. Fourth, to examine whether unilateral or bilateral tDCS would be superior for producing this improvement, participants would complete three separate tDCS rounds: one where sham stimulation was administered; another where tDCS is directed at the left hemisphere, with the anode electrode near the left DLPFC; versus another condition where anode electrodes were placed over the left and right frontal lobes respectively. In this manner, we could examine if connectivity in the frontoparietal network (FPN) could benefit from the stimulation of as many network nodes as possible, or whether the stimulation delivered to a single hemisphere was sufficient for producing similar results despite being lateraled. For the reasons previously noted, we expected bilateral stimulation would be superior. In summary, we hypothesized that only participants who performed below normal on the N-Back at baseline would also demonstrate improvements on the MMSE, especially when receiving bilateral tDCS.

## Methods

### Materials and equipment

#### Materials used.

**Fig 1:**
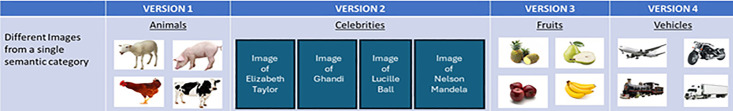
Stimuli used for the different versions of the N-Back. In Round 1, version 1 was the trained version of the N-Back, while version 2 remained untrained. In Round 2, version 2 was practiced (trained) for the first time, and version 3 served as the untrained version during evaluations. In round 3, version 3 was practiced, and version 4 was untrained.

The N-Back task is a cognitive test used to measure working memory and attention, where a series of stimuli (e.g., letters or shapes) are presented one at a time. The participant’s job is to indicate when the current stimulus matches the one presented N steps back in the sequence. The value of N can vary, such as 1-back, 2-back, etc., but in the present study, N was always a one-back. We chose the N-Back task for this study due to several strategic advantages. First, the N-Back task is known to engage working memory [54]. Second, its design minimizes practice effects through inherent randomization of stimuli, a method shown to limit strategy development and learning effects in n-back tasks [55,56]. Third, the task’s format allows for easy modification by altering the stimuli array, making it adaptable to different formats. Fourth, its requirement for constant decision-making enables participants to engage in extended practice sessions, with the number of decision points programmable to fill the designated 20-minute window or any other desired duration. Inspired by the N-Back created by Basak and O’Connell [57], we created four versions of this task that would be used as the trained and untrained versions across the different study rounds, functionally the same but consisting of different stimuli (Fig 1). In all cases, four different images from a particular semantic category (animals, celebrities, fruits, vehicles) were presented. In Round 1, animals were the version of the N-Back practiced by participants, while celebrities were unpracticed and only presented during evaluations without feedback. In the second round, the version showing celebrity faces was practiced by participants for the first time, thus becoming the trained items for that round, while fruits were the version untrained and evaluation-only. Finally, in the third round, the fruit version was practiced, while vehicles served as the untrained version that was only shown in evaluations without feedback.

To ensure each version of the N-Back task would produce comparable response times (RTs), a group of 20 elderly individuals (12 females, ages 65–80), was recruited to provide normative data on the task. Exclusion criteria included the presence of mental illness or history of stroke, and diagnosis of dementia, determined by on-site MoCA tests scoring 27 or above out of 30. This group was chosen to serve as a representative sample of the age group targeted for this task. Norming results showed each version of the N-Back using different stimuli sets produced equivalent RTs, allowing for an average RT to be calculated (mean = 912.12 ms, SD = 30.41 ms). This normative range served as a benchmark for interpreting if the RTs produced by a targeted study participant at baseline were slower than normal.

#### tDCS equipment and modeling.

To investigate the hypothesis that stimulating both hemispheres with tDCS would be more effective than stimulating only the left hemisphere, our study required a different tDCS application method than in previous studies. Instead of using a single anodal electrode to administer 2 mA tDCS to the left hemisphere, we utilized a splitter box that could administer 2 mA tDCS to two anode electrodes placed on the left and right anterior regions of the scalp (Fp1 and Fp2, which represent the left and right frontal pole respectively) with a cathode electrode over the occipital lobe (Oz). This montage configuration was chosen to maximize blinding and ensure that participants always experienced the same electrode setup. Furthermore, a computer simulation model (HD-Explorer by Soterix Medical) predicted that this arrangement would produce a current flowing through both lateral prefrontal cortices when directed to a single cathodal electrode placed posteriorly over the occipital notch. HD-Explorer is an advanced software tool used for simulating brain current flow in transcranial electrical stimulation (tES) techniques like tDCS and HD-tDCS. The accuracy of HD-Explorer has been validated in both human and non-human primate subjects, ensuring its reliability for research and clinical applications [58]. Meanwhile, the tDCS machines produced by Soterix Medical, are approved for clinical and research applications [59].

In this study, when the right anterior anode electrode is deactivated, the setup represents a standard unilateral tDCS application targeting solely the left frontal cortex and its underlying subcortical areas. For this reason, although we had the option to place an additional cathode electrode over the occipital region, we opted for a single cathode to distinctly attribute any differences between unilateral and bilateral montages to the inclusion of an active right anterior anode (at Fp2). In the bilateral scenario, each anodal electrode received a 2 mA current, culminating in a total of 4 mA entering the brain and exiting via the lone cathodal electrode. To match this current intensity in the left frontal cortex in the unilateral tDCS condition, we administered 4 mA tDCS solely at Fp1. Another approach could have been to set the unilateral condition at 2 mA, distributing 1 mA to each hemisphere. However, given that the established norm for tDCS clinical efficacy is 2 mA, we were concerned that 1 mA per hemisphere might be insufficient for a favorable outcome. Consequently, we decided to provide 2 mA tDCS to each hemisphere in the bilateral condition and make the unilateral intensity be 4 mA, ensuring that the stimulation levels in both conditions had comparable intensity.

**Fig 2:**
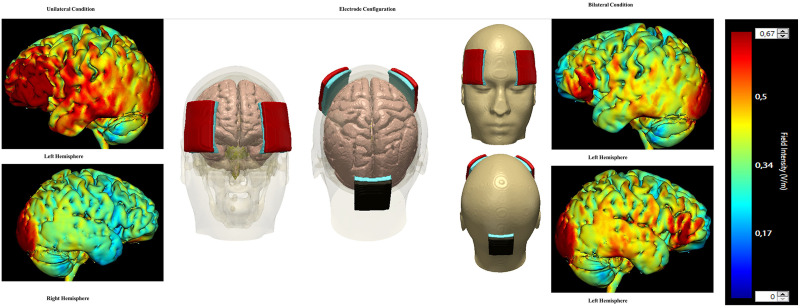
Electrode and Stap Configuration for the unilateral and bilateral tDCS conditions. Settings on the splitter box and tDCS machine determine if the stimulation reached one or two anode electrodes, and whether the stimulation itself was active or sham. The Left and right panels display the activation predictions for the unilateral and bilateral conditions respectively; images generated by HD-Explore (Soterix).

The electrode arrangement and anticipated field distributions (Fig 2) are displayed employing a color gradient to denote intensity levels across brain regions—red signifies a peak intensity of 0.67 mA. These visualizations derive from simulations on an Adult Male 1 model, using a 93-electrode setup to approximate a 5x7 tDCS array. We introduced a flexible design capable of simulating intensities exceeding 2 mA. Specifically, we modeled a scenario where 4 mA enters through an anode positioned over Fp1 and exits via a cathode at the back of the head (Oz).

This montage (Fig 2) is predicted to produce heightened cortical activity in the left hemisphere, whereas the right hemisphere will display minimal activation, indicated by a predominance of green. Conversely, in the bilateral setup, we simulated 2 mA targeting an anode over Fp1, and an additional 2 mA entering another anode at Fp2 in the right anterior hemisphere. The cathode, as in the unilateral scenario, was set to facilitate a 4 mA exit. This arrangement, demonstrated in Fig 2, is predicted to produce a more balanced activation across both hemispheres, with increased activity (indicated by more red areas) in the right hemisphere. Thus, while the unilateral condition predicts stronger activation in the left hemisphere, the bilateral condition predicts a more uniform and moderate level of activity across both hemispheres.

The electrode placement used for this study (Fig 2) was the same in all conditions vis-à-vis set-up. To ensure consistent placement of electrodes across all montages, electrodes measuring 5 by 7 cm were vertically placed on the participants’ scalp in designated areas based on the EEG 10–20 measurement system [60]. Prior to placing the electrodes, sponges were soaked using syringes with 25 ml of saline (0.09%) and then slipped onto the electrodes, with the dot roughly in the center of the sponge. Rubber straps were used to secure the sponges in place. All electrodes were connected to a Soterix splitter box, which controlled the active electrodes, and connected to a Soterix 1x1 conventional tDCS machine, which determined the duration and intensity of the stimulation, as well as whether the stimulation would be real or sham. The stimulation duration in all conditions was 20 minutes. In particular, the splitter box was primarily responsible for determining which electrodes were active, with the right frontal anodal electrode being off in the unilateral condition and on in the bilateral condition. The protocol ensured blinding because participants always experienced the same electrode setup.

### Blinding techniques

Neither the evaluators nor the participants were aware if the tDCS they received during the training sessions was bilateral, unilateral, or sham. Therefore, the study was effectively double-blind. To ensure that participants were properly blinded to the study condition, we employed an adaptive ramp-up procedure that gradually increased the intensity level of tDCS by 0.5 mA every 30 seconds until reaching an intensity level of 4 mA. This technique is inspired by the methods used by Khadka et al. [61], who also gradually increased intensity rather than a standard fast ramp-up. Because there was a potential risk that using the adaptive ramp-up method only during 4 mA administration might reveal to participants that they were receiving the 4 mA condition, which could undermine the blinding effect, we added the additional step to the ramp-up procedure to all stimulation conditions. More specifically, all sessions began with an adaptive ramp-up to 4 mA tDCS, and after the participant reported tolerating 30 seconds of 4 mA tDCS, a research assistant restarted the machine and applied a standard tDCS ramp-up to that session’s intended intensity level (i.e., sham, 2 mA, or 4 mA). This technique ensured that the experience for all participants was the same, regardless of the stimulation condition, and eliminated the risk of revealing the 4 mA condition. By first habituating participants to the 4 mA intensity level, we expected that they would be better able to tolerate the stimulation and that blinding would be properly maintained throughout the study across the different tDCS rounds.

For those rounds that involved sham stimulation, which was applied after the adaptive 4 mA ramp-up, the machine first ramped up to 4 mA before quickly ramping back down to 0, delivering no stimulation for the remainder of the session, except to ramp up to 4 mA and then back to zero during the final minute. This activation pattern was designed to mimic the habituation effect experienced during real tDCS, where participants quickly habituate to the sensation of stimulation, with perception being most prominent at the beginning of a session when the charge ramps up, or at the end of the session when the charge fades to extinction. The changes in intensity during these phases are what participants perceive. Thus, the sham stimulation in this study was designed to simulate the initial and final phases of real tDCS by replicating the ramp-up and ramp-down of active tDCS stimulation. As sham stimulation occurred after participants had habituated to 4 mA tDCS, we expected participants would be unable to differentiate between sham, 2 mA, or 4 mA tDCS.

To monitor adverse events, participants were verbally administered an adverse events questionnaire at the end of each tDCS session to monitor side effects beyond the common tingling or redness, such as headaches or other adverse reactions. Starting from the first session and continuing after each subsequent session, participants were also asked whether they experienced any side effects the previous evening. The questionnaire assessed headache, facial/dental/eye pain, neck pain, hearing problems, stiffness, seizures, syncope, nausea, mood changes, and other issues.

### Healthy older adult control group

Twenty individuals aged 60–85 living in Montreal, Quebec, Canada completed all four versions of the N-Back task in a single session, each consisting of 28 responses per version. To account for order and practice effects, the different versions were counter-balanced across participants. 12 participants were female, and all participants obtained a score above 26 on the MoCA and the MMSE the day their RTs on the N-Back were completed. Considering the simplicity of a one-back for healthy adults, accuracy was expected to be high (if not perfect) across all versions. Indeed, other than occasional “accidents” quickly reported by controls, there were virtually no judgement mistakes for any of the versions. Crucially, we also found that RTs across the conditions were similar, in that controls took similar amounts of time to respond on the N-Back task regardless of the version presented, which was an average of 912.12 ms, with a standard deviation of 30.41 ms. Thus, a cutoff of 988.15ms (mean RT plus 2.5 standard deviations) was taken as the cut-off point for the normal performance range. Participants with brain damage who showed a mean RT greater than 988.15ms, would be those considered abnormally slow on the task.

### tDCS participants

This study was conducted across two research labs in Montréal and Toronto. Participants were recruited from local Memory Clinics, where they had already received a formal diagnosis of AD or FTD. Specifically, participants were recruited from the Jewish General Hospital in Montréal and the Sam and Ida Ross Memory Clinic at Baycrest Health Sciences, or the Anne and Allan Bank Centre for Clinical Research Trials, in Toronto. Recruitment took place from February 3^rd^, 2018, until January 1^st^, 2024; however, the study itself was placed on hold from March 2019 to January 2022 as all research halted during the covid pandemic. Participants provided written consent during the enrollment process. Minors were not included in the study. Both research labs carried out the same study design, using identical materials and tDCS equipment. The research ethics board of each institution approved the study (Montreal: CODIM-MBM-16–025; Toronto: 16–025). To be eligible to participate in the study, participants needed to be proficient in either English or French, although linguistic background was not expected to have a significant impact on the results as the N-Back is a non-verbal task and the MMSE has both English and French versions. Participants had to demonstrate objective cognitive impairment, scoring below 26 on the Montreal Cognitive Assessment (MoCA) [62], and impairment in executive function operationalized as scoring 3 or less out of 5 on the visuospatial/executive sub-section of the MoCA. The MoCA was issued in the person’s primary language. All participants were required to demonstrate capacity to provide informed consent prior to enrollment. As part of the pre-study screening process, each participant met with one of the primary investigators, who explained the study’s purpose, procedures, risks, and requirements. This discussion allowed the investigator to assess whether the participant clearly understood the study and was able to make an informed decision. Only individuals who demonstrated such understanding were enrolled. This consent procedure was reviewed and approved by the Research Ethics Boards at each institution.

There were no medication exclusions, but participants were asked to report any medication changes related to dementia or cognitive health that occurred during the study. Exclusion criteria included the presence of non-degenerative neurological disorders (e.g., stroke). Although we hypothesized that participants who performed in the normal range of the N-Back would fail to demonstrate improvements coinciding with a tDCS effect, we nevertheless chose to enroll them on the ethical grounds that these participants might nevertheless benefit from participation due to presenting with general cognitive impairments. In short, these participants would be individuals who despite demonstrating cognitive impairments consistent with dementia, would nevertheless perform well on the N-Back task administered to them. We aimed to recruit a large enough sample of participants that we could later retrospectively allocate to two dichotomous groups: normal and below-normal N-Back RTs. In this manner, we could simultaneously compare bilateral and unilateral tDCS to sham for improving cognition but also check for the replication of the RISE principle in our data.

Ultimately, we screened 30 individuals and enrolled 27 individuals diagnosed with either AD or FTD. However, one individual had severe disease progression over the course of the study, while two participants maintained significantly improved N-Back RTs after their first rounds; therefore, the data from these individuals was omitted. Thus, the results presented reflect 24 participants (17 with AD, 7 with FTD; 12 females, 12 males). An equal number of participants (8) completed each treatment arm: a pre-determined order of stimulation rounds that ensured stimulation condition order was counterbalanced across participants. No medication changes were reported.

We wished to determine whether each participant’s RT was within the normal range, or slower than normal (i.e., impaired). The normative data derived from the mean RT of a comparable normal control group (see section titled “healthy older adult control group”) was used as a reference for establishing a normal range cut-off score (baseline cut-off score of >988.15 ms, with scores above this rated abnormal). For each AD or FTD participant, a baseline comparison score was calculated as the average of six N-Back reaction times, derived from the trained and untrained tasks completed at the participant’s first evaluation in each of the three rounds. Fortuitously, 12 out of 24 participants were found to have baseline responses slower than normal., defined as an RT greater than the control mean RT plus 2.5 standard deviations (i.e., >988.15 ms). The remaining participants had RTs that were within a range comparable to controls (i.e., normal range). This established two participant subgroups: a normal RTs subgroup, and a subgroup of participants with slower than normal baseline RTs. Table 1 presents these participants and categorizes them based on their baseline comparison score, and the subsequent RT group in which they were analyzed. Additional demographic information is also presented, including the MMSE score obtained at enrollment. Groups did not differ significantly in education or mmse scores; however, participants with slower-than-normal response times on the N-Back task at baseline were on average 419.38 ms slower on the N-Back task (t (22) = 7.84, effect size = 3.34) and were around seven years older (*t*(22) = 1.92, *d* = 0.82). As previously discussed, prolonged response times have been linked *t*o underlying brain impairment and reduced cognitive function [63–66], indicating that these individuals may be particularly suitable candidates for tDCS—an idea supported by the RISE principle.

**Table 1:** Demographics and baseline scores.

Participant Number	Diagnosis (AD/FTD)	Age	Sex (M/F)	Education	MMSE at Enrollment	Average N-Back Score across Baseline Evaluations (ms)
**Participants with Normal Response Times on the N-Back**						
1	AD	72-76	F	13	26	502.03
2	FTD	59-63	M	12	17	628.88
3	AD	83-87	M	12	20	646.61
4	AD	57-61	M	16	21	660.62
5	FTD	86-90	M	16	23	762.52
6	AD	77-81	M	15	21	821.86
7	FTD	80-84	F	13	19	843.08
8	AD	67-71	F	18	26	858.41
9	AD	62-66	F	14	27	876.48
10	AD	73-77	M	12	23	919.65
11	FTD	86-90	F	16	27	972.46
12	FTD	55-59	F	13	16	975.94
**AVERAGE**	**7 AD**	**73.42**	**6 F**	**14.17**	**22.17**	**789.05 ms**
**Participants with Slower than Normal Response Times on the N-Back**						
13	AD	74-78	F	18	21	1023.3
14	FTD	79-83	M	11	24	1089.01
15	AD	81-85	M	11	20	1122.71
16	AD	91-95	F	12	21	1152.84
17	AD	92-96	F	16	19	1162.32
18	FTD	77-81	M	16	26	1186.82
19	AD	71-75	M	16	22	1207.3
20	AD	72-76	M	17	27	1247.51
21	AD	74-78	F	12	26	1268.89
22	AD	73-77	M	16	29	1295.99
23	AD	75-79	F	20	23	1339.65
24	AD	88-92	F	8	24	1404.78
**AVERAGE**	**10 AD**	**80.92**	**6 F**	**14.42**	**23.5**	**1208.43 ms**

### Study design

The study aimed to compare the effectiveness of different montages in improving N-Back RTs and if those improvements would translate into general cognition improvements. Participants underwent three stimulation conditions, with each condition consisting of a different montage – bilateral, unilateral, and sham. To ensure an unbiased comparison, the order of montages was counterbalanced across participants using stratified randomization. Participants were given a wash-out period of at least 2 months between each round to allow for the dissipation of tDCS effects and to return to baseline performance. A timeline of these rounds over the course is presented in Table 2.

**Table 2:** Timeline of the three tDCS rounds over 27 weeks.

Week	1	2	3	4	5
**Round 1**	Treatment A + N-Back Practice				Evaluation
			
**Week**	**12**	**13**	**14**	**15**	**16**
**Round 2**	Treatment B + N-Back Practice				Evaluation
			
**Week**	**23**	**24**	**25**	**26**	**27**
**Round 3**	Treatment C + N-Back Practice				Evaluation

Each round involved three weeks of training sessions and evaluations: Week 1: baseline evaluation on Tuesday, stimulation sessions on Thursday and Friday; Week 2: stimulation sessions on Monday, Tuesday, Wednesday, Thursday, and Friday; Week 3: stimulation sessions on Monday and Tuesday, second evaluation with stimulation on Wednesday. Two weeks after the second evaluation session, participants were given the same evaluation, but without tDCS. The schedule for the different evaluations and stimulation sessions is displayed in Table 3.

**Table 3:** Timeline of stimulation and evaluation sessions for a single round.

Week	Monday	Tuesday	Wednesday	Thursday	Friday
**1**		Baseline Evaluation before tDCS		tDCS Session	tDCS Session
**2**	tDCS Session	tDCS Session	tDCS Session	tDCS Session	tDCS Session
**3**	tDCS Session	tDCS Session	Evaluation with tDCS		
**4**					
**5**			Evaluation without tDCS		
**11**			Evaluation without tDCS		

### Training session and evaluation protocols

Except for the stimulation condition and the specific stimuli used in the N-Back task, all rounds adhered to the same protocol. Throughout each training session, lasting a total of 20 minutes (i.e., the duration of the stimulation), participants were assigned to a predetermined stimulation condition (sham, bilateral, or unilateral), while practicing the N-back task on a computer. Each round involved a different version of the N-back task, using a unique set of images. The presentation of stimuli occurred on laptops utilizing Presentation software (Version 18.0, Neurobehavioral Systems, Inc., Berkeley, CA, USA; http://www.neurobs.com).

During each evaluation, two versions of the N-Back task were administered: one that would be practiced during that round’s sessions, and another untrained version. The versions used were determined by the round number (1, 2, or 3). This approach allowed us to explore the different impacts of tDCS on skill improvement, specifically learning (measured by improvement on the trained version) and generalization (measured by improvement on the untrained version), which would indicate a more widespread improvement. Additionally, to confirm improvement in general cognitive function, participants were also given the MMSE in their primary language (English or French) during evaluations.

### Carry-over and disease progression effects

The study is complicated by potential carry-over effects and disease progression effects. Carry-over effects may result in participants maintaining a persistent electrically stimulated neuronal benefit after completing a round of active tDCS stimulation, rather than having a wash-out effect during the 2-month gap between stimulation rounds. Disease progression may make certain participants untestable or affect their baseline performance on subsequent rounds. To address these issues, we calculated the RT mean and standard deviation of each participant at baseline for trained and untrained items to exclude any participant with a mean that changed more than 1.5 standard deviations from their own baseline mean from one round to the next. We also removed any participants who displayed a clear and observable decline after returning for a subsequent tDCS round, defined as declining more than 5 points on the MoCA. We can report that one individual had severe disease progression over the course of the study, while two participants maintained significantly improved N-Back RTs after their first rounds. The data from these participants was excluded from analyses.

### Data cleaning

To ensure that RTs for the N-Back task in each evaluation were accurate, we removed RTs associated with incorrect responses [67], as well as those shorter than 500 ms or longer than 20,000 ms. Additionally, we only included data from participants who produced at least 20 correct responses (out of 27); a criterion that all participants met. We then calculated the mean and standard deviation of the remaining RTs for each participant to identify individual outlier responses. Specifically, an RT was considered an outlier if it exceeded the mean plus or minus three standard deviations, following the recommendation by Van Selst and Jolicoeur [68] for the number of responses produced by participants. We removed these outliers from each participant’s set of RTs, resulting in a new mean and standard deviation that represented the participant’s average RT on the N-Back. Ultimately, less than 5% of responses were removed.

For missing data, we imputed the last known response time (RT) from the participant’s previous evaluation. This approach was applied in two cases for the unilateral condition: (1) participant 3’s second evaluation, where response data was lost due to a technical failure, and (2) participant 7’s third evaluation, which could not be completed due to their inability to attend the scheduled session. Participant 11 in the unilateral condition produced identical response times across two separate evaluations. These values were independently recorded and verified, and no data duplication or entry error was identified. All three participants were part of the normal response time group.

### Statistical analysis

Statistical analyses were performed using SAS software version 9.4 (SAS Institute Inc., 2013). The study employed a mixed design, incorporating within-subjects factors (stimulation conditions: bilateral, unilateral, sham) and a between-subjects factor (baseline reaction times: normal vs. slower-than-normal). The independent variable was the type of stimulation condition, while the dependent variables included reaction times (RTs) on the N-Back task and MMSE scores. A repeated-measures ANOVA was conducted to assess the effects of Condition (bilateral, unilateral, and sham) and Time (eval 1, eval 2, eval 3) as within-subject factors, with Baseline RTs (normal, slower-than-normal) as a between-subjects factor. When significant interactions or main effects were detected, paired one-sided t-tests were performed to compare RTs across the three stimulation conditions at specific time points. All statistical tests were one-tailed, and Holm–Bonferroni corrections were applied to account for multiple comparisons (i.e., two follow-up comparisons to baseline within each stimulation condition).

## Results

### N-Back results

We analyzed the results separately for the trained and untrained versions of the N-Back task, using a similar approach for each. Specifically, we conducted a repeated-measures ANOVA with Condition (unilateral, bilateral, sham) and Time (Eval 1, Eval 2, Eval 3) as within-subject factors, and Baseline (normal, slower than normal) as a between-subject factor.

For the trained items, Mauchly’s test of sphericity was significant for Time (χ² = 8.46, *p* < .05); therefore, Huynh-Feldt corrections were applied when interpreting effects involving this variable. Only the main effect of Time was significant, *F*(1.66, 36.54) = 26.89, *p* < .001, η² = .550, indicating that participants improved on the version they practiced, with no significant differences between stimulation conditions. For untrained items, Mauchly’s test of sphericity was again significant for Time (χ² = 9.18, *p* < .05), and Huynh-Feldt corrections were applied. A significant Time × Condition × Baseline interaction was found, *F*(3.75, 82.55) = 2.95, *p* = .028, η² = .118.

We repeated the repeated measures as before for untrained items, but separately for each sub-group: participants in the normal RTs group (n = 12), and participants with a slower than normal baseline RTs (n = 12), For the normal RTs group, Mauchly’s test of sphericity was significant for the condition variable (χ² = 8.47, *p* < .05) and the condition x time variable ((χ² = 19.16, **p* *< .05); therefore, Huynh-Feldt corrections were applied. The time variable was significant *F* (1.66, 18.30) = 6.41, *p* = .010, η² = .37), as well as the condition variable *F* (1.37, 15.01) = 5.38, **p* *= .026, η² = .33, were significant, but not the condition x time variable, suggesting there was no change over time related to the stimulation condition (i.e., active vs. sham tDCS). In contrast, for participants with slower than normal baseline RTs, there was no violation of sphericity, and a significant condition x time effect was found (*F* (4, 44) = 4.12; *p* = .006, η² = .272), suggesting that improvement in this group was dependent on the form of stimulation administered.

**Fig 3:**
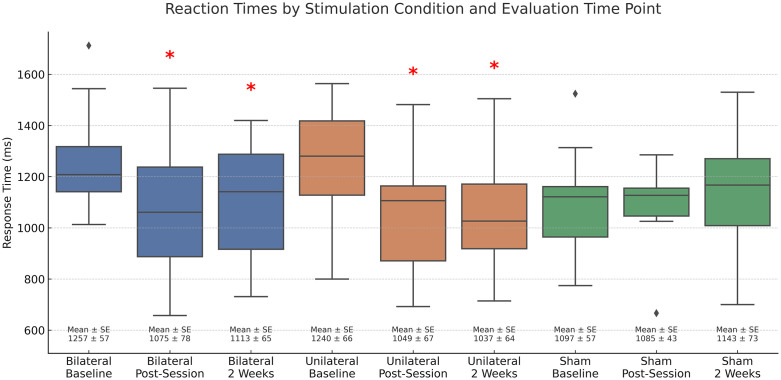
Response Time (ms) Changes across Stimulation Conditions and Time Points for the Untrained Version of the N-Back in participants with slower than normal response times. Asterisks (*) indicate a statistically significant reduction in response time compared to baseline (p < .05). Diamonds represent outliers. “Bilateral” and “Unilateral” refer to active stimulation conditions, while “Sham” indicates the placebo condition. Error bars represent the standard error of the mean for each group and time point.

To further investigate the significant condition × time interaction indicated by the repeated measures ANOVA, we conducted paired t-tests for untrained items to compare reaction times (RTs) collected at baseline to those collected at the second evaluation (one day after the final training session) and again two weeks later, across the three stimulation conditions (unilateral, bilateral, sham). A Holm–Bonferroni correction was applied to account for the six planned comparisons. Consistent with the repeated-measures ANOVA results, the bilateral condition showed a significant RT reduction at the final session (t(11) = 3.021, p = .006, Holm threshold = .0125, d = 0.878). Two weeks later, the improvement persisted, with RTs still significantly faster than baseline (t(11) = 2.513, p = .014, Holm threshold = .0167, d = 0.73). In the unilateral condition, RTs were significantly faster at both follow-ups: at the final session (t(11) = 4.183, p < .001, Holm threshold = .0083, d = 1.21) and two weeks later (t(11) = 5.80, p < .001, Holm threshold = .01, d = 1.68); all comparisons surpassing their respective Holm–Bonferroni thresholds. In contrast, no significant changes in RTs were observed in the sham condition at either follow-up (p > .20). Participants were approximately 14.45% faster than baseline at the second evaluation in the bilateral condition and 15.42% faster in the unilateral condition, with sustained improvements two weeks later—16.41% faster in the unilateral condition and 11.40% faster in the bilateral condition (Fig 3).

### MMSE results

To determine whether N-Back improvements generalized to broader cognitive outcomes, we analyzed MMSE scores at baseline, immediately following the final stimulation session, and again two weeks later using planned paired-samples t-tests. Due to the small sample size (n = 12 per group), results should be interpreted with caution; however, the slower RT group findings are supported by prior evidence of stimulation effects, whereas the faster RT group’s MMSE results are exploratory.

**Fig 4:**
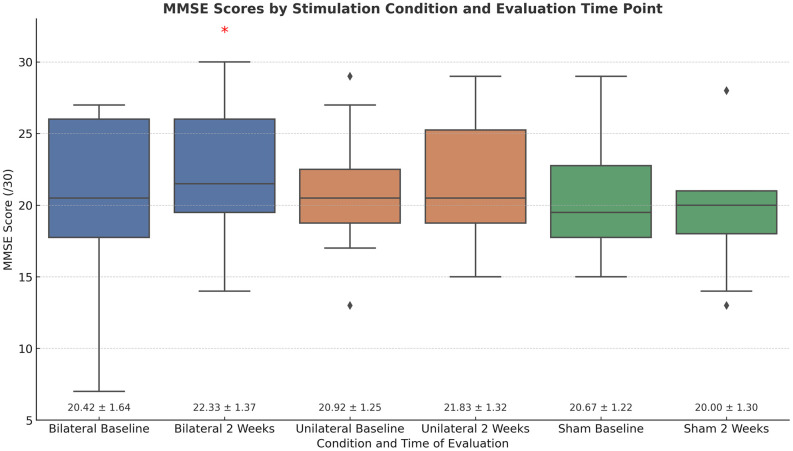
MMSE Score Changes in Participants with Slower-than-Normal Response Times: Baseline versus Two Weeks Post-Stimulation across Stimulation Conditions. Boxplots display Mini-Mental State Examination (MMSE) scores at baseline and two weeks later for each stimulation condition: Bilateral, Unilateral, and Sham. Asterisks (*) indicate a statistically significant improvement relative to baseline (p < .05). Diamonds represent outliers. Mean ± standard error (SE) values are presented beneath each condition. Error bars reflect variability within each group.

In the slower RT group, bilateral tDCS was associated with a significant increase in MMSE scores two weeks post-intervention (t(11) = 2.19, p < .05, d = 0.63), with no significant changes observed in the unilateral or sham conditions. As this was a planned follow-up based on observed N-Back effects, no correction for multiple comparisons was applied. In contrast, the normal RT group did not show any significant MMSE changes across conditions (all ps > .05). While MMSE results were examined in both groups, only data from the slower RT group are shown in Fig 4, as this was the only group to demonstrate a significant stimulation × time interaction on the N-Back task. These findings support the interpretation that generalization to broader cognitive function may be specific to the slower RT group—and specifically to when they received bilateral stimulation.

### A secondary analysis of trained items

**Fig 5:**
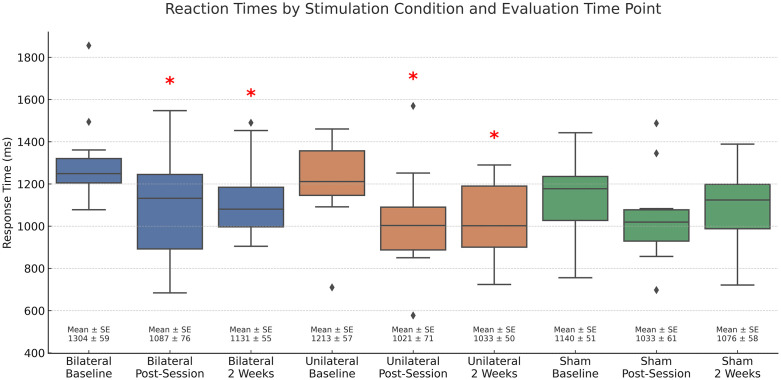
Change in Response Time (ms) Across Stimulation Conditions and Time Points for the Trained Version of the N-Back in participants with slower than normal response times. Asterisks (*) indicate a statistically significant reduction in response time compared to baseline (p < .05). Diamonds represent outliers. “Bilateral” and “Unilateral” refer to active stimulation conditions, while “Sham” indicates the placebo condition. Error bars represent the standard error of the mean for each group and time point.

In this study, we investigated the impact of a particular intervention on a specific outcome variable and used a repeated-measures ANOVA to determine whether there were significant differences between the stimulus conditions for trained and untrained items. The results of the ANOVA showed that there was no overall significant effect (*p* > .05) for trained items. However, we decided to conduct paired t-tests to compare the RTs produced by the group with slower than normal RTs for trained items, as t-tests are sensitive to detect differences between specific conditions, which is what we wanted to explore further. This decision was also based on the positive results found for untrained items and the MMSE. The t-tests revealed that participants in this group demonstrated a significant improvement in both the bilateral and unilateral stimulation conditions, during the final session and two weeks later, with no significant improvement in the sham condition. Specifically, there was a statistically significant reduction in RT in the bilateral condition at the final session (t(11) = 3.21, p = .004, Holm threshold = .0125, d = 0.94) and two weeks later (t(11) = 3.88, p = .001, Holm threshold = .0083, d = 1.13). Similarly, RTs in the unilateral condition were significantly faster at the final session (t(11) = 3.19, p = .004, Holm threshold = .0167, d = 0.93) and two weeks later (t(11) = 3.51, p = .002, Holm threshold = .0100, d = 1.01). These results remained significant after applying Holm–Bonferroni correction for six comparisons. Consistent with the findings for untrained items, participants performed approximately 15.86% faster than baseline immediately after the final training session and 14.86% faster two weeks later in the unilateral condition, while participants in the bilateral condition were 16.62% faster at the final session and 13.29% faster two weeks later. These percentages (Fig 5) reflect the mean percent change in RT relative to baseline values in each condition.

### Adverse events and blinding results

During a debriefing session at the end of the study, we asked participants if they could identify in which round they had received sham stimulation. Most participants (16) reported simply “no”; five participants reported they thought all rounds had been real despite being told one would be placebo, and the remaining participants all incorrectly identified the sham round. Side-effects were standard feelings of tingles during the initial minutes of stimulation, and temporary redness that dissipated after a few hours. No other adverse effects were observed.

## Discussion

Our study compared the efficacy of bilateral and unilateral tDCS for enhancing working memory and general cognitive function in individuals with dementia (AD and FTD). Based on the R.I.S.E. principle—which posits that Reduced Initials lead to Superior Enhancements—we has four central predictions: 1) There would be greater improvement on the N-Back when practice occurred during active tDCS; 2) Only participants impaired in the task would should an improvement related to the stimulation condition; 3) For the active tDCS rounds, N-Back response times would improve most when bilaterial rater than unilateral stimulation is administered; and 4) Participants improvement on the N-Back task would also be accompanied by an improvement in general cognition. In summary, we expected that the broader cortical coverage from bilateral tDCS would outperform unilateral stimulation and sham in improving both RTs and MMSE scores.

Findings partially supported these predictions. Consistent with our first two predictions, only those participants who were impaired on the N-Back task demonstrated greater improvement when active tDCS was administered, but this was true for both active montages (Fig 3), with effect sizes larger for the unilateral condition; thus, contradicting our third prediction. However, consistent with our fourth prediction, improvement on the N-Back task was accompanied by improved general cognition, as indexed by MMSE scores, and only in the bilateral stimulation condition MMSE scores (Fig 5). These findings reinforce the idea that tDCS effects are contingent upon baseline cognitive state and task engagement—core to the R.I.S.E. principle—and align with established neurorehabilitation frameworks that emphasize individualized, task-dependent intervention strategies. The R.I.S.E. principle shares conceptual ground with models of state-dependent plasticity, whereby interventions yield the most robust effects when neural systems are under-engaged or hypoactive [69]. In line with this, the observed improvements may be mediated by neuroplastic mechanisms such as enhanced resting-state connectivity and the modulation of impaired oscillatory activity—particularly within the theta and gamma bands implicated in working memory. While speculative, these mechanisms are consistent with prior studies showing that tDCS can normalize aberrant rhythms and boost network efficiency [70,71]. Our results also support the importance of concurrent task engagement; the N-Back task may have acted as cognitive scaffolding, enhancing the specificity and magnitude of tDCS effects. This interpretation aligns with meta-analytic findings [72] and empirical studies [73] showing that cognitive benefits from tDCS are most pronounced when combined with training, while tDCS alone yields negligible effects. These findings highlight that neuromodulation is not inherently efficacious; rather, its success often depends on synergy with cognitive activation.

Regarding the frontoparietal network (FPN), both bilateral and unilateral stimulation improved RTs, suggesting that focal engagement of the left DLPFC may be sufficient to activate broader network dynamics. This observation echoes neuroimaging studies showing high interconnectivity within the FPN [74], where local excitation can propagate through functional hubs. For example, Claaß et al. [75] used concurrent tDCS-fMRI to show that anodal stimulation over the DLPFC modulates resting-state frontoparietal connectivity, supporting the notion that stimulation effects extend across broader cognitive networks. Indeed, only bilateral tDCS led to MMSE improvements, implying that broader hemispheric stimulation may be necessary for more generalized cognitive gains, potentially due to more widespread synaptic modulation across degraded networks in dementia. This difference may also reflect known physiological asymmetries and functional specializations. For instance, although the left DLPFC plays a critical role in executive function, bilateral stimulation may recruit additional regions in the right hemisphere important for broader attentional control, error monitoring, or global cognitive coordination [69,76,77]. Moreover, electric field modeling studies suggest that bilateral montages can lead to greater overall current penetration and distribution across prefrontal and parietal cortices, potentially supporting more widespread network plasticity [78,79]. This is consistent with findings from Nissim et al. [80], who showed that bilateral DLPFC activation during working memory tasks was associated with better performance in older adults, highlighting the potential benefits of bilateral engagement in aging populations.

Some protocols—particularly in rTMS—have employed asymmetrical stimulation where stimulation is excitatory for the left hemisphere, while simultaneously dampening the activity of the corresponding right hemisphere [81,82]. However, our findings suggest that for individuals with dementia, bilateral tDCS may be more effective in driving general cognitive improvements, possibly due to the need for more widespread synaptic engagement across degraded networks. In dementia, neural atrophy and disconnection are not limited to focal regions but often involve diffuse disruptions across large-scale networks, including the frontoparietal and default mode networks. Bilateral stimulation may therefore be necessary to sufficiently activate these distributed systems, enhance interhemispheric connectivity, and promote compensatory recruitment of homologous or adjacent regions. This broader engagement may help overcome localized dysfunction by leveraging residual network integrity and facilitating global coordination required for complex cognitive tasks. Future work should clarify how stimulation symmetry and lateralization interact with specific cognitive and affective targets, particularly in neurodegenerative populations.

Limitations of the present study must also be acknowledged. The sample size was modest, which limits statistical power and increases the risk of both Type I and Type II errors. With a small sample, true effects may go undetected, and observed effects may be less stable or exaggerated due to sampling variability. This is particularly important when interpreting the significance of within-subject comparisons and interactions (Figs 3–5). Additionally, participants exhibited diagnostic and cognitive heterogeneity, which may have introduced additional variance. While we attempted to account for baseline performance, future studies should aim to recruit larger, more stratified samples to improve generalizability and statistical precision. Long-term follow-up will also be important to determine the durability and clinical relevance of observed effects. Finally, the absence of neuroimaging data limits our ability to validate the proposed mechanisms of action and to link behavioral changes directly to neurophysiological alterations.

In conclusion, bilateral tDCS over the DLPFC, paired with a working memory task, led to improvements in both task-specific and general cognition, but only in those participants with lower baseline performance. These findings support the R.I.S.E. principle and the need for personalized, task-coupled stimulation strategies in neurorehabilitation. A better understanding of stimulation mechanisms and the role of baseline state will be crucial in refining tDCS applications for clinical use.

## Supporting information

S1 Data Reproducibility Data SI Modified. (XLSX)
